# Equipping missionaries for the mission: capacity building initiatives and the decentralised planning in Jharkhand, India

**DOI:** 10.1186/1753-6561-6-S1-O7

**Published:** 2012-01-16

**Authors:** Suranjeen Prasad, VR Raman, Haldhar Mahto, Rajan Kumar, Dinesh Jagtap, Shampa Roy, Alexander Kerketta

**Affiliations:** 1 Public Health Resource Network, New Delhi, India; 2 Department of Health and Family Welfare, Government of Jharkhand, India; 3 National Health System Resource Centre, New Delhi, India

## Introduction

In 2006, even with the external support, most of the districts in Jharkhand state were unable to do health planning beyond an initial prospective planning. The key impediment was the lack of capacity to analyse the health situation in their districts and develop strategies and propose budgets to implement these. The Public Health Resource Network (PHRN) in partnership with the National Health Systems Resource Centre (NHSRC) developed a training curriculum and a fast-track capacity building programme in 2006 to address this gap.

Jharkhand state adopted this training programme and trained selected officials from all districts in several batches beginning in the year 2008. Social workers from various civil society organisations were also trained on district health planning through a parallel distance-learning programme by PHRN. The trained personnel from both health department (n=155) and civil society groups (n=85) supported the district programme management unit in preparing district health action plan during November 2009 to March 2010.

This study was done in order to assess the significance of the PHRN-led capacity building in enhancing the individual and institutional technical capacities at the district level. Study aimed to understand how the capacity building enhanced the district level planning and management of health service deliveries under the National Rural Health Mission (NRHM).

## Methods

A case study on district health planning processes was prepared through a desk review of reports, review of district health plan documents, focus group discussions with the district teams, and interviews with stakeholders.

## Results

We found that at least 10 of the 24 districts developed in-house capacity to take forward the health planning processes at district level as a result of the capacity building programme. This Improved planning capacity at district level has enhanced the enthusiasm for decentralised planning replacing the earlier notion that planning is a normative compulsion under NRHM.

The situation analysis and planning was better in the districts where the selection of personnel for the training was done. Involvement of civil society members improved compliance to the decentralized planning process. The capacity building intervention improved the focus on inclusive planning and equity through special plans for vulnerable areas and groups.

All districts were able to prepare and submit their action plans on their own for the first time, with limited guidance and appraisal inputs from experts at the state level. Most of the plan proposals from the districts were incorporated in the state project implementation plan for 2010-11. The subsequent 2011-12 planning process has adopted the same strategy.

## Discussion

There is a need to improve the competence of the district team for planning and management of public health systems through systematic capacity building. The selection of personnel for such capacity building programmes is crucial for the success of the programme. Even though several years have passed since the launch of NRHM, and there have been some capacity-building inputs, there are gaps especially in the regions where this is most needed. The key challenge is how these improvements can be institutionalised and maintained.

Also, there is a need to ensure resource allocation based on the needs indicated by the district health plans. In absence of this, the enthusiasm created by the capacity building initiatives may diminish.

